# Preservation of functionality, immunophenotype, and recovery of HIV RNA from PBMCs cryopreserved for more than 20 years

**DOI:** 10.3389/fimmu.2024.1382711

**Published:** 2024-08-16

**Authors:** Wayne B. Dyer, Kazuo Suzuki, Angelique Levert, Mitchell Starr, Andrew R. Lloyd, John J. Zaunders

**Affiliations:** ^1^ Strategy & Growth, Australian Red Cross Lifeblood, Sydney, NSW, Australia; ^2^ The Kirby Institute, University of NSW, Sydney, NSW, Australia; ^3^ NSW State Reference Laboratory for HIV, Centre for Applied Medical Research, St Vincent’s Hospital, Sydney, NSW, Australia

**Keywords:** cryopreservation, biobank, viability, immunophenotyping, HIV reactivation, T cell subsets, memory T cells, T cell function

## Abstract

**Background:**

Many research laboratories have long-term repositories of cryopreserved peripheral blood mononuclear cells (PBMC), which are costly to maintain but are of uncertain utility for immunological studies after decades in storage. This study investigated preservation of cell surface phenotypes and *in-vitro* functional capacity of PBMC from viraemic HIV+ patients and healthy seronegative control subjects, after more than 20 years of cryopreservation.

**Methods:**

PBMC were assessed by 18-colour flow cytometry for major lymphocyte subsets within T, B, NK, and dendritic cells and monocytes. Markers of T-cell differentiation and activation were compared with original immunophenotyping performed in 1995/1996 on fresh blood at the time of collection. Functionality of PBMC was assessed by culture with influenza antigen or polyclonal T-cell activation, to measure upregulation of activation-induced CD25 and CD134 (OX40) on CD4 T cells and cytokine production at day 2, and proliferative CD25+ CD4 blasts at day 7. RNA was extracted from cultures containing proliferating CD4+ blast cells, and intracellular HIV RNA was measured using short amplicons for both the Double R and pol region pi code assays, whereas long 4-kbp amplicons were sequenced.

**Results:**

All major lymphocyte and T-cell subpopulations were conserved after long-term cryostorage, except for decreased proportions of activated CD38+HLA-DR+ CD4 and CD8 T cells in PBMC from HIV+ patients. Otherwise, differences in T-cell subpopulations between recent and long-term cryopreserved PBMC primarily reflected donor age-associated or HIV infection-associated effects on phenotypes. Proportions of naïve, memory, and effector subsets of T cells from thawed PBMC correlated with results from the original flow cytometric analysis of respective fresh blood samples. Antigen-specific and polyclonal T-cell responses were readily detected in cryopreserved PBMC from HIV+ patients and healthy control donors. Intracellular HIV RNA quantitation by pi code assay correlated with original plasma viral RNA load results. Full-length intracellular and supernatant-derived amplicons were generated from 5/12 donors, and sequences were ≥80% wild-type, consistent with replication competence.

**Conclusions:**

This unique study provides strong rationale and validity for using well-maintained biorepositories to support immunovirological research even decades after collection.

## Introduction

Cryopreservation of clinical peripheral blood specimens is routine for stem cell transplantation and cellular therapy programs and frequently required in vaccine and immunotherapy trials. Short-to-medium-term storage of peripheral blood mononuclear cells (PBMC) is well established in research protocols to allow assessment of immunological responses in centralised laboratories to avoid site and operator-based variation and to enable simultaneous comparison of longitu1dinally collected specimens in clinical trials and cohort studies, particularly in subjects with known outcomes. Long-term biobanking is also commonly undertaken to support future strategic research. For instance, in relation to blood-borne virus (BBV) infections, stored specimens from historical patient cohorts, such as HIV-infected patients sampled prior to the availability of highly active antiretroviral treatments (HAART) or patients with acute hepatitis C sampled prior to the advent of the rapidly curative direct-acting antiviral treatments, could provide insights into immunological response patterns relative to current patients. Similarly, to determine the role of historical protective immunity against a current disease such as SARS CoV-2, access to PBMC stored before the major outbreaks and widespread immunisation programs are required. However, the unknown viability and functional quality of such historical collections may cast doubt on the value of these investigations and raise concerns of the financial burden associated with maintaining these collections.

The useful lifespan of a biorepository and the factors influencing sample degradation are debated topics. The functional quality of cryopreserved PBMC samples is influenced by many confounding variables, including blood sample collection and shipment conditions (1, 2), timely and proficient processing of blood samples according to specified methods and reagents (3–7), and training and support via relevant quality assurance programs (4, 8). PBMC quality parameters are also influenced by thawing procedures (9, 10), and cryogenic temperature excursions during specimen retrieval and handling (5, 11). Regardless of these logistical, processing, and handling variables, the overall value of a well-curated and stored PBMC repository depends on the time frame for which ideally cryopreserved and stored PBMC retain adequate viability, leukocyte subset representation, and functional capacity, to support research objectives of end users.

Previous studies have reported well-preserved PBMC function capacity after long-term cryopreservation, defined as 12 months (7, 12, 13), 7 years (3), and over 10 years (6). By contrast, other studies have reported a functional decline in cryopreserved cellular therapy products within 3 years (14), or after only 1 year in a research collection (15). It remains unclear to what extent these varied durability outcomes were attributable to limitations in the logistical, processing, and handling of the samples, as opposed to slow deterioration in the functional integrity of PBMC stored long term in liquid or vapour phase nitrogen below the glass transition point of water (−135°C), which is the point at which virtually all biological functions stop (16).

A biorepository of PBMC from HIV+ and HCV+ patient cohorts and healthy control populations was established with regular sample collection and storage between 1994 and 2005, when the repository was incorporated into the ongoing Immunovirology Research Network (IVRN; https://ach4.org.au/immunovirology-research-network-ivrn/). The IVRN includes an Australia-wide network of laboratories with skills in separation and storage of PBMC, a biannual quality assurance program for this activity, and a centralised biorepository, all underpinning provision of high-quality PBMC samples to support strategic Australian immunovirology research in relation to BBV infections. The IVRN biorepository allowed comparison of viability, subset representation, and immune function of PBMC cryopreserved for greater than 20 years compared with the original fresh whole blood analysis and to samples cryopreserved for less than 1 year. The findings demonstrate high viability, preserved leukocyte populations and T-cell subsets, functional capacity, and intact viral RNA from archived HIV+ patient PBMC, demonstrating the utility of such samples for current research.

## Methods

### Ethical approval

Access to specimens from historical biorepository specimen collections and contemporary specimen collections was approved by the UNSW Human Research Ethics Committee (HC200777), and St Vincent’s Hospital Human Research Ethics Committee (HREC/13/SVH/145 and HREC/10/SVH/130).

### Participants and PBMC specimens

Long-term cryopreserved PBMC from HIV-positive (n=12) and healthy uninfected control donors (n=20), originally collected between 1995 and 1997 (17) were used for this study. These samples were subjected to whole blood immunophenotyping on the fresh samples at the time of collection (18). In addition, recently cryopreserved PBMC (<12 months; n=20) from healthy anonymous laboratory staff, collected as control samples for the IVRN quality assurance program, were used. Fresh PBMC were also obtained from healthy volunteers, as previously described (19, 20).

### PBMC fractionation and cryopreservation

Whole blood was centrifuged at 1,000 g for 10 min, plasma was removed for storage, and then buffy coat cells were removed and diluted in serum-free RPMI medium, underlaid with Ficoll-Paque Premium (Sigma-Aldrich, Melbourne, Australia) and then centrifuged at 700g for 20 min with the brake off. PBMC were harvested and washed once in RPMI and then once in RPMI with 10% pooled human serum (in-house reagent). After counting, PBMC were resuspended in chilled cryopreservation medium made fresh from RPMI with 10% cell culture grade DMSO (Sigma-Aldrich) and 20% pooled human serum, dispensed into chilled cryovials, and then immediately processed in a controlled-rate freezer and transferred into liquid nitrogen.

### PBMC thawing and viability assessment

PBMC vials were transferred from vapour-phase nitrogen storage on dry ice, rapidly thawed in a 37°C water bath only until a small ice pellet remained, diluted incrementally to 14 ml with RPMI/10% foetal bovine serum (FBS; CSL, Australia) within 30 s, and centrifuged at 300g for 8 min. After a second wash in RPMI/10% FBS, the PBMC were resuspended in 0.5 ml phosphate-buffered saline (PBS) and treated with DNase 1 Solution (0.1 mg/ml; STEMCELL Technologies, Vancouver, Canada) for 15 min at room temperature, according to the manufacturer’s directions, and then washed in RPMI/10% FBS. The DNase treatment was used to remove free DNA released from dead cells, which caused loss of viable cells by adherence to cell clumps during thawing and washing. Recently cryopreserved PBMC were not treated with DNase. PBMC (lymphocytes and monocytes) were counted in a Coulter ActDiff haematology analyser (Beckman Coulter, Brea, CA). Viability was assessed by Trypan Blue exclusion counted manually in a haemocytometer.

### PBMC lineage flow panels

One million PBMC were resuspended in 0.5 ml PBS with 1.5 µl Aqua viability exclusion dye and incubated for 30 min at room temperature, and then quenched with 1 ml FBS, washed in medium, resuspended in 100 µl, and divided into two tubes, before adding antibody master mixes for lineage and T-cell panels (**Supplementary Table 1**). After a 15-min incubation, cells were washed with 2 ml PBS and resuspended in 250 µl of 1% paraformaldehyde in PBS (PFA/PBS) analysis within 2 h on a five-laser LSRFortessa flow cytometer (BD Biosciences, Franklin Lakes, NJ). For each set of experiments, cytometer settings were controlled using Cytometer Setup and Tracking Beads (BD Biosciences) and a daily compensation matrix was generated using compensation beads (BD Biosciences) coated with each antibody, as previously described (19). Freeze–thaw-degraded PBMC stained with Aqua viability dye were used for compensation in the Aqua channel.

### Antigen-specific CD4 T-cell memory measured by OX40 activation-induced marker assay, day 7 proliferation assay, and cytokine release

To assess CD4 T-cell memory function in historical PBMC samples, two million PBMC were suspended in 1,200 µl RPMI with 10% pooled human serum (in-house reagent) and divided into six wells of a 96-well culture plate, and duplicate wells were incubated at 37°C (i) with no addition (negative control); (ii) supplemented with 2 µl influenza vaccine (Influvac Tetra, 2018 formulation, Mylan Health, Sydney, Australia)7; and (iii) supplemented with 2 µl anti-CD3/28/2 (STEMCELL Technologies; positive control), respectively. After 2 days of incubation, 80 µl medium was removed from one set of wells for cytokine assay (see below), cells were resuspended, and 40 µl of the cell suspension was added to the OX40 assay antibody cocktail containing anti-CD3, -CD4, -CD25, and -CD134 monoclonal antibodies (**Supplementary Table 1**), incubated for 20 min, washed in PBS, and resuspended in 250 µl PFA/PBS for flow cytometric identification of CD25+CD134+ antigen-specific CD4 T cells, as previously described (20, 21). A positive OX40 response was defined as ≥0.2% of CD4 T cells, as previously described (22).

Supernatants were collected at 48 h from each of the three separate OX40 activation-induced marker (AIM) cultures set up for each PBMC sample, as described above. Supernatants were stored at −30°C and batch tested for each of the following cytokines IL-1β, TNF-a, IFN-g, IL-17, IL-10, and IL-22, using a multiplex cytometric bead assay (LEGENDplex, BioLegend, San Diego, CA, USA) according to the manufacturer’s directions and analysed on a five-laser Fortessa flow cytometer (BD Biosciences). Concentrations of cytokines were determined from standard curves using Qognit data analysis software (BioLegend) according to the manufacturer’s directions. The limit of detection for each cytokine was ≤1 pg/ml, and the upper limit of each assay was 10,000 pg/ml (except IFN-g: 18,000 pg/ml; IL-22: 15,000 pg/ml; and IL-17: 12,000 pg/ml).

The remaining wells were cultured for a further 5 days, and proliferating CD4 T cells at day 7 were identified as CD25high blast cells (enlarged on Forward Scatter) as previously described (23).

### Viral nucleic acid extraction from activated T cells, and HIV-1 RNA amplification and detection

After a 7-day activation and proliferation of PBMC cultured with anti-CD3/28/2 (described above), HIV-1 RNA was extracted from the cells, using the Maxwell RSC automated extraction platform, with the Maxwell RSC Simply RNA Tissue kit (Promega Corporation, Madison, WI, USA) according to the manufacturer’s recommendations. Also, total nucleic acid (TNA) was purified from the culture supernatants using the Maxwell RSC Viral Total Nucleic Acid kit (Promega).

HIV-1 RNA was amplified using primers and probes targeting the HIV-1 LTR R region, as previously described (24, 25). In addition, two sets of primers were used for the Pol region, Set-15 forward AAAAGAAAAGGGGGGATTGGG and reverse TACTGCCCCTTCACCTTTCCA (positions 4785 to 4976, 191 bp), plus Set-17 forward GGGGGTACAGTGCAGG and reverse TGTATTACYACTGCCCCTTCACCTTT (positions 4804 to 4984, 180 bp), and probe AAAAAAAAAAAAAAATTTGGAAAGGACCAGC. The PCR was performed using Luna Probe One-Step RT-qPCR 4X Mix with UDG (New England Biolabs, Ipswich, MA, USA), and the following cycling conditions: a reverse transcriptase step at 55°C for 10 min, one denaturation step at 95°C for 2 min, followed by 35 cycles of (95°C for 13 s, 62°C for 40 s). The amplicons were analysed using our previously described piCode method (Suzuki et al, AIDS 2019). Quantification of HIV-1 copy number was determined with a standard curve generated with a HIV-1 gBlock gene fragment (Integrated DNA Technology, Coralville, Iowa, USA): 0.64-2000 HIV-1 copies/µl. The HIV-1 copy number was normalised per one million of WBCs.

### Nanopore sequencing

Amplicons from long transcripts of HIV RNA, covering *gag-pol* region, were prepared with One-Step RT-PCR followed by nested PCR using the Superscript IV One-Step RT-PCR System (Thermo Fisher Scientific, Waltham, MA, USA). RT-PCR was performed using outer primers, forward TGGGTGCGAGAGCGTC and reverse TACTGCCCCTTCACCTTTCCA (4185 bp), and the following conditions: 45°C for 20 min followed by 98°C for 2 min, 50 cycles of (98°C for 10 s, 68°C for 45 s, 72°C for 2 min 15 s), and a final elongation at 72°C for 5 min. The nested PCR was performed using inner primers, forward GAGATGGGTGCGAGAGCGTCA and reverse ACTGTAYCCCCCAATCCCCC (4,027 bp), and PCR cycling conditions as follows: 98°C for 2 min followed by 50 cycles of (98°C for 10 s, 62°C for 45 s, 72°C for 2 min 15 s), and a final elongation at 72°C for 5 min.

The amplicons were purified using Agencourt AMPure XP (Beckman Coulter, Brea, CA, USA), and the concentration was measured with the Qubit 4 Fluorometer and Qubit 1X dsDNA HS Assay Kit (Thermo Fisher Scientific). DNA libraries for nanopore sequencing were prepared using Native Barcoding Kit 96 V14 (SQK-NBD114.96) following the protocol Ligation sequencing amplicons, and Native Barcoding Kit 24 V14 (NBA_9168_v114_revH_15Sep2022) provided by Oxford Nanopore Technology (ONT, Oxford, UK). The DNA amplicons were end-repaired, barcoded with unique adapter indexes, and pooled, and long fragment buffer was used for the final wash. The concentration of the prepared DNA library was measured with the Qubit before loading into the port of a R10.4.1 flow cell (ONT). The sequencing run was performed for 1 h, with fast-base-calling, in a MinION Mk1C device and MinKNOW software (ONT). The sequences were evaluated by reference to the Stanford HIV Drug Resistance Database, and mutations were reported using a Mutation Detection Threshold of 20%.

### Statistical analysis

Summary statistics were expressed as mean and SD. Data were assessed for normality to guide choice of t-test vs. Mann–Whitney, and ANOVA vs. Kruskal–Wallis tests for comparisons between groups, ANOVA vs. Friedman tests for paired multiple comparisons, and Spearman correlations between thawed PBMC and historical whole blood immunophenotyping data.

## Results

### Donor groups and PBMC sample details

Donor and sample details for each group, including age at donation, time in cryostorage, viability, and recovery, are shown in **Table 1**. The ages of the healthy control subjects at collection for their long-term cryopreserved samples were greater than for the long-term cryopreserved HIV+ patients (p=0.0319). The donor age for the recently cryopreserved PBMC was estimated from a subset of known donors in this predominantly anonymous donor group. All HIV+ patients were infected for >10 years at the time of sample collection, none had received antiretroviral treatment, most were asymptomatic and classified as HIV “slow-progressors” (CD4 T-cell counts (mean ± SD): 609 ± 327), and all were viraemic (plasma viral load: 101,542 ± 250,058 copies/ml). Viability of thawed PBMC decreased slightly after long-term cryopreservation (p=0.0612); however, only 5 of the 31 long-term cryopreserved PBMC had viability below 80%. The recovery of cells after long-term cryopreservation was higher than the recently cryopreserved PBMC, likely confounded by potential differences in counting methods used over time.

**Table 1:** Group characteristics of study participants.

	Fresh blood healthy controls	Recent cryo healthy controls	Long-term cryo healthy controls	Long-term cryo viraemic HIV+	^‡^p-values
**Number**	16	20	19	12	NA
**Donor age**	46.3 ± 12.6	*43.4 ± 11.9	59.9 ± 15.8	47.2 ± 10.2	0.0319
**Cryo (years)**	N/A	0.29 ± 0.26	24.0 ± 0.26	26.9 ± 0.4	<0.0001
**Viability**	N/A	94.2 ± 3.5%	90.8 ± 6.7%	87.7 ± 8.8%	0.0612
**Recovery**	N/A	78.9 ± 22.0%	141 ± 34.8%	135 ± 65.7%	<0.0001

Data expressed as mean ± SD. NA, not applicable. *Estimate based on a subset of donors (n=6); age data not available from other anonymous donors. ^‡^Kruskal–Wallis test comparing recent with long-term cryopreserved PBMC groups.

### Leukocyte lineages in PBMC are preserved after long-term cryopreservation

**Figure 1:**
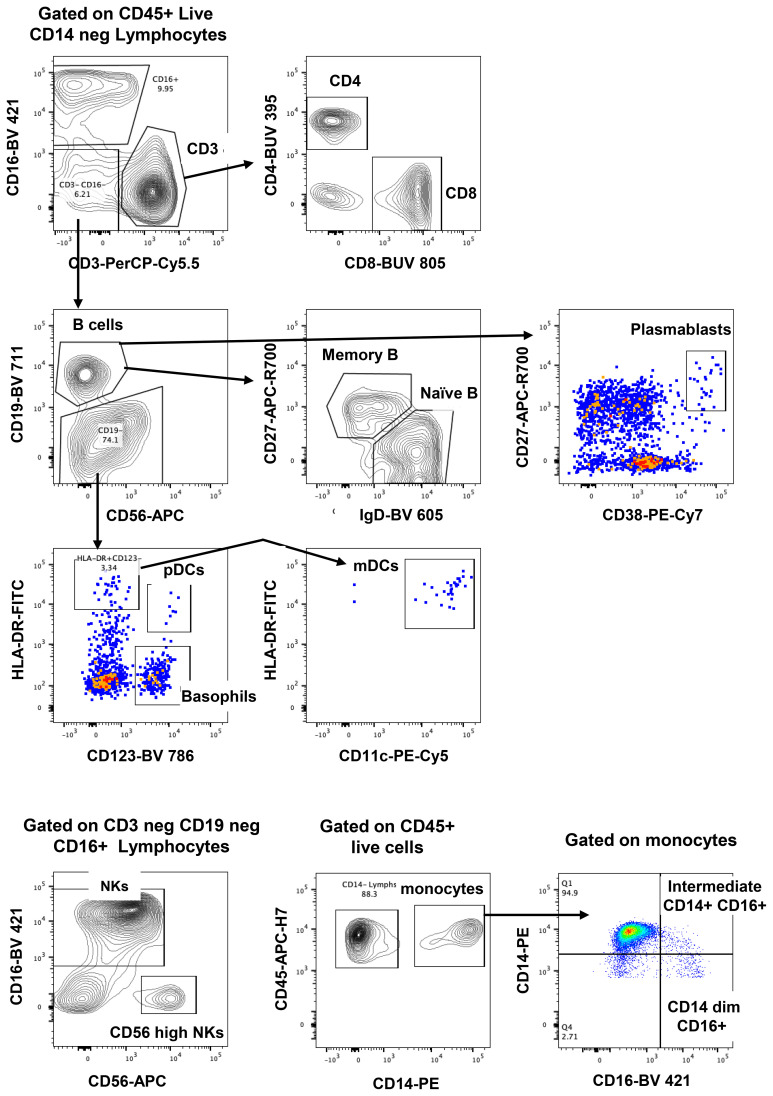
Representative flowplots showing immunophenotype gating for major subpopulations of PBMC.

**Figure 2:**
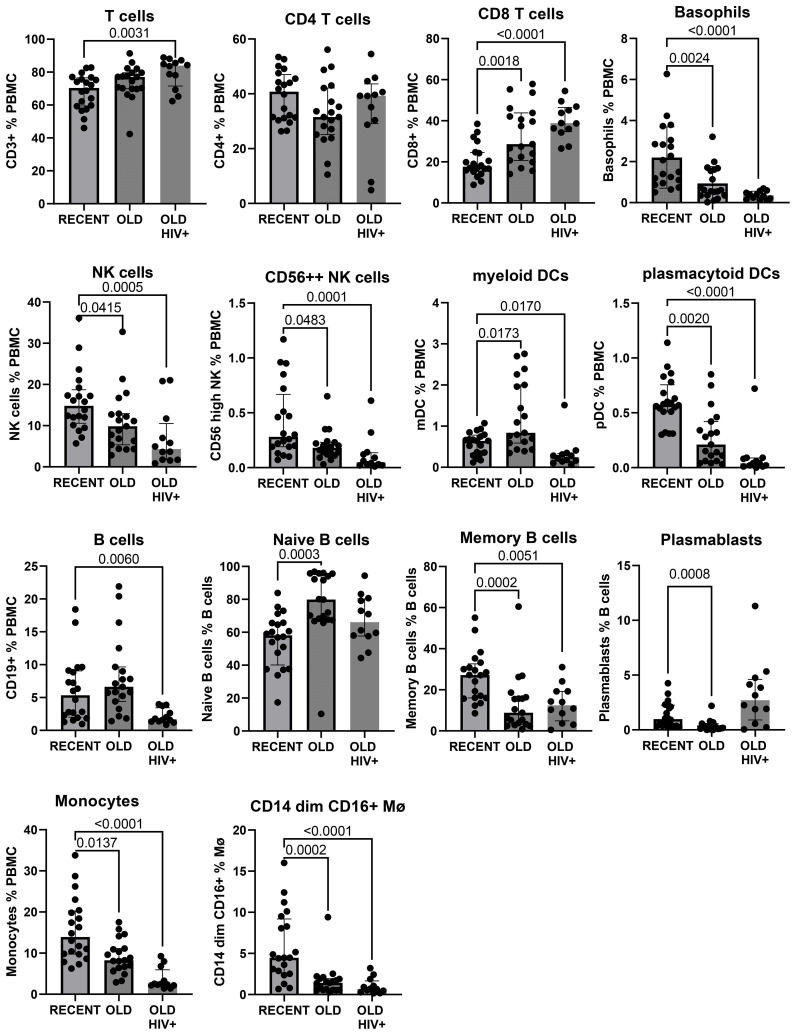
Major subpopulations of PBMC in recently cryopreserved healthy controls compared with long-term cryopreserved healthy control and viraemic HIV+ patient PBMC.

The gating strategy to enumerate major CD45+ subpopulations from long-term cryopreserved PBMC is shown in **Figure 1**. Phenotyping of the major subsets of PBMC, including T cells, B cells, NK cells, dendritic cells, monocytes, and basophils, confirmed that these subsets were all present in specimens stored for >20 years (**Figure 2**). While significant differences were observed in most subpopulations, they were most probably attributable to differences in donor age between groups, in particular reduced pDCs (26) and CD56++ NK cells (27), or findings typical of untreated HIV-1 infection. Reduced numbers of monocytes, particularly the CD16+ subset, may be due to long-term cryopreservation, because these cells are reportedly increased in the elderly (28, 29). Nevertheless, all major populations were represented in the long-term cryopreserved PBMC samples.

### T-cell subsets in PBMC were preserved after long-term cryopreservation

**Figure 3:**
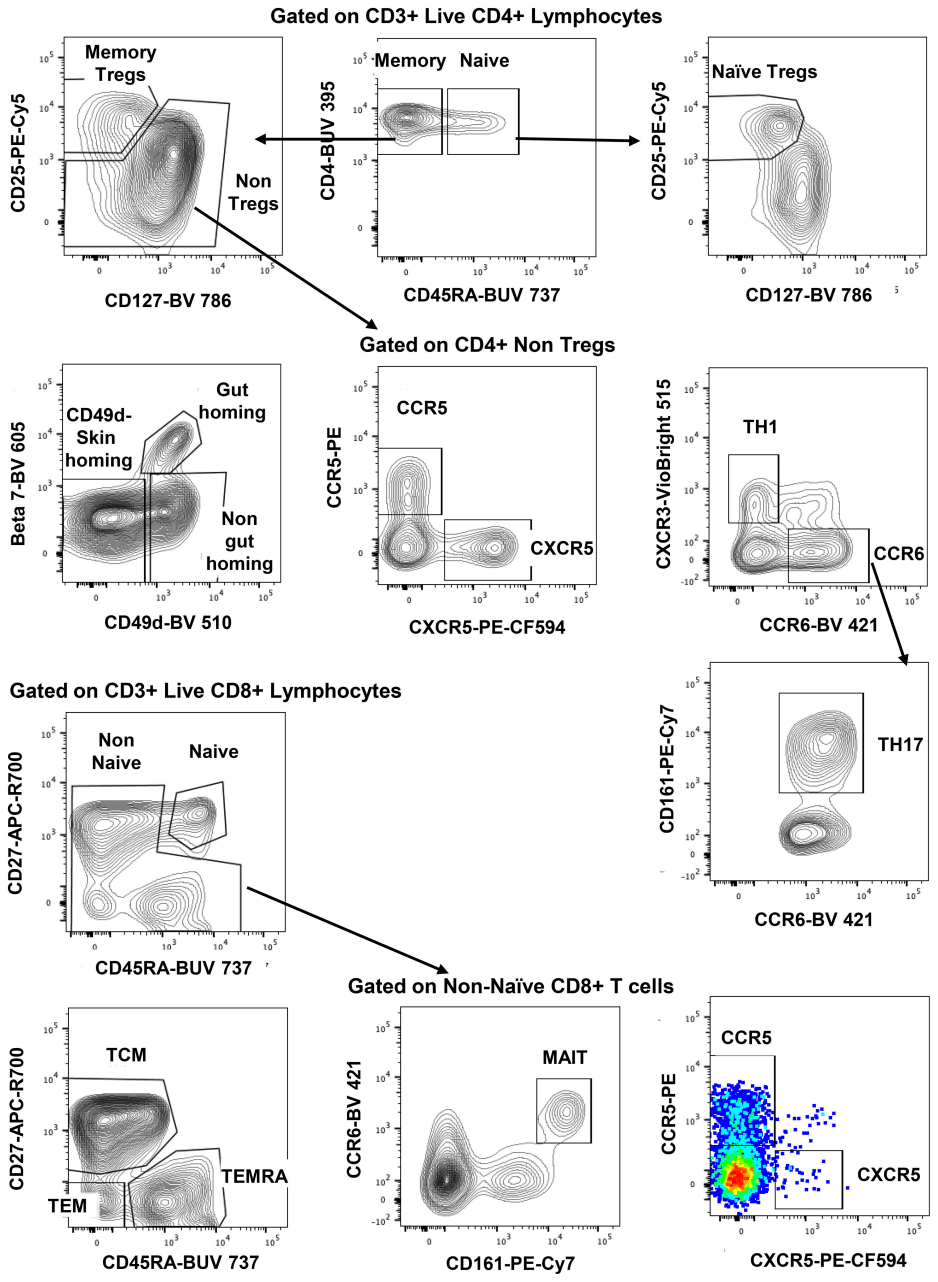
Representative flowplots showing immunophenotype gating for major subsets of CD4+ and CD8+ T cells in thawed PBMC samples.

**Figure 4:**
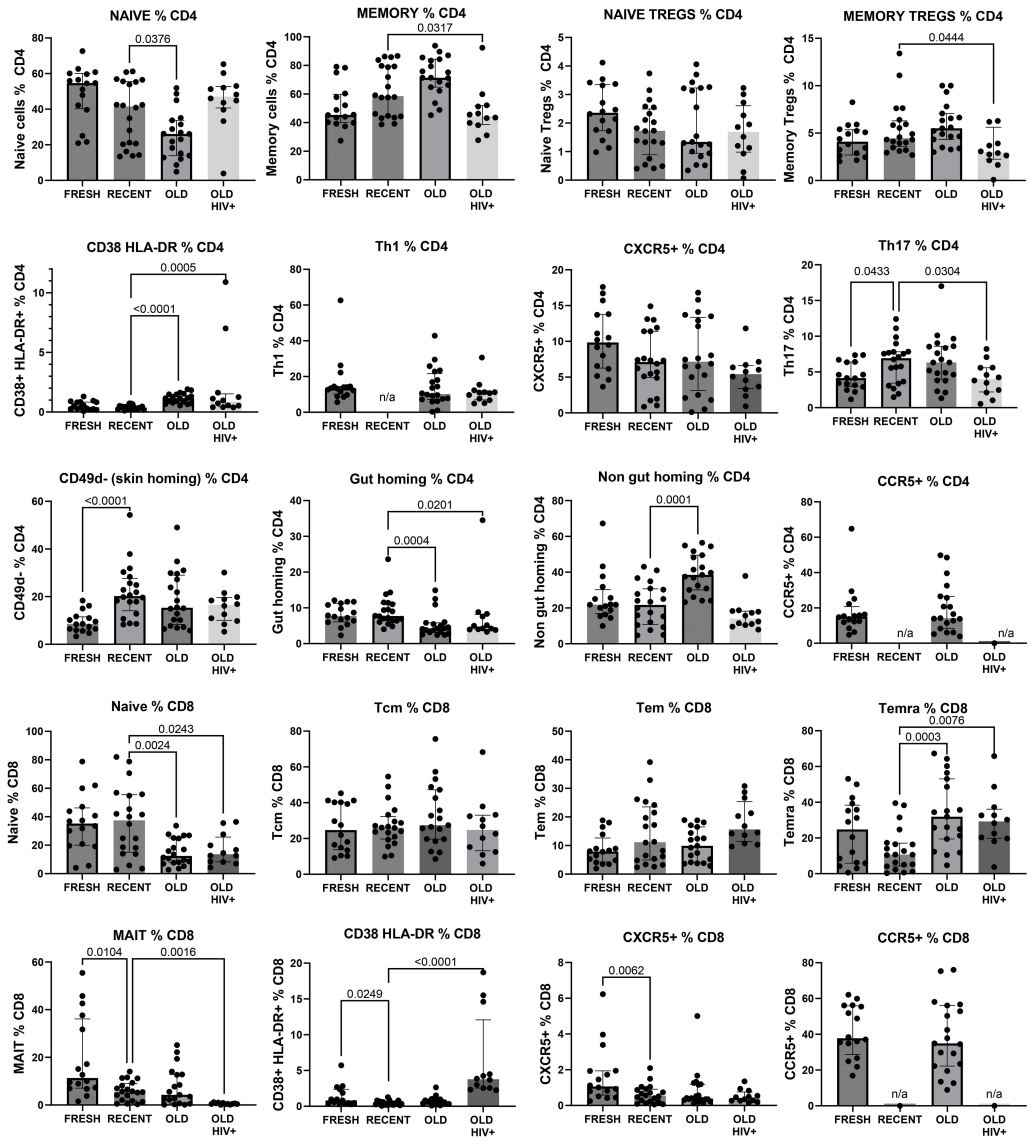
Major CD4+ and CD8+ T-cell subsets in recently cryopreserved healthy control PBMC, compared with fresh PBMC, long-term cryopreserved healthy control PBMC, and long-term cryopreserved viraemic HIV+ patient PBMC.

The gating strategy for CD4+ and CD8+ T-cell subset phenotyping, shown in **Figure 3**, was performed on recent and long-term cryopreserved PBMC, as well as fresh blood samples. All T-cell subsets were represented in long-term cryopreserved PBMC (**Figure 4**). Some differences between long-term and recently cryopreserved PBMC were again most probably associated with donor age and/or HIV-1 infection status, not storage time. These included reduced naïve CD4 and CD8 T cells and naïve TREGs, increased memory and activated CD4 and CD8 T cells, and increased memory CD4 TREGs and increased CD8 TEMRA cells, all previously reported in older and/or HIV+ subjects (30, 31) (see also Discussion). However, we also observed that long-term and even recent cryopreservation appears to decrease CD49d expression, and therefore long-term stored specimens had the lowest frequencies of gut-homing phenotype CD4 T cells. Expression of CCR5, the predominant HIV-1 co-receptor, appeared to be well maintained long-term. Comparisons between fresh blood and cryopreserved PBMC suggested that cryopreservation may reduce proportions of activated MAIT and CXCR5+ subsets of CD4 and CD8 T cells (**Figure 4**).

### Correlation between historical whole blood and thawed PBMC immunophenotyping data from the same blood draw

**Figure 5:**
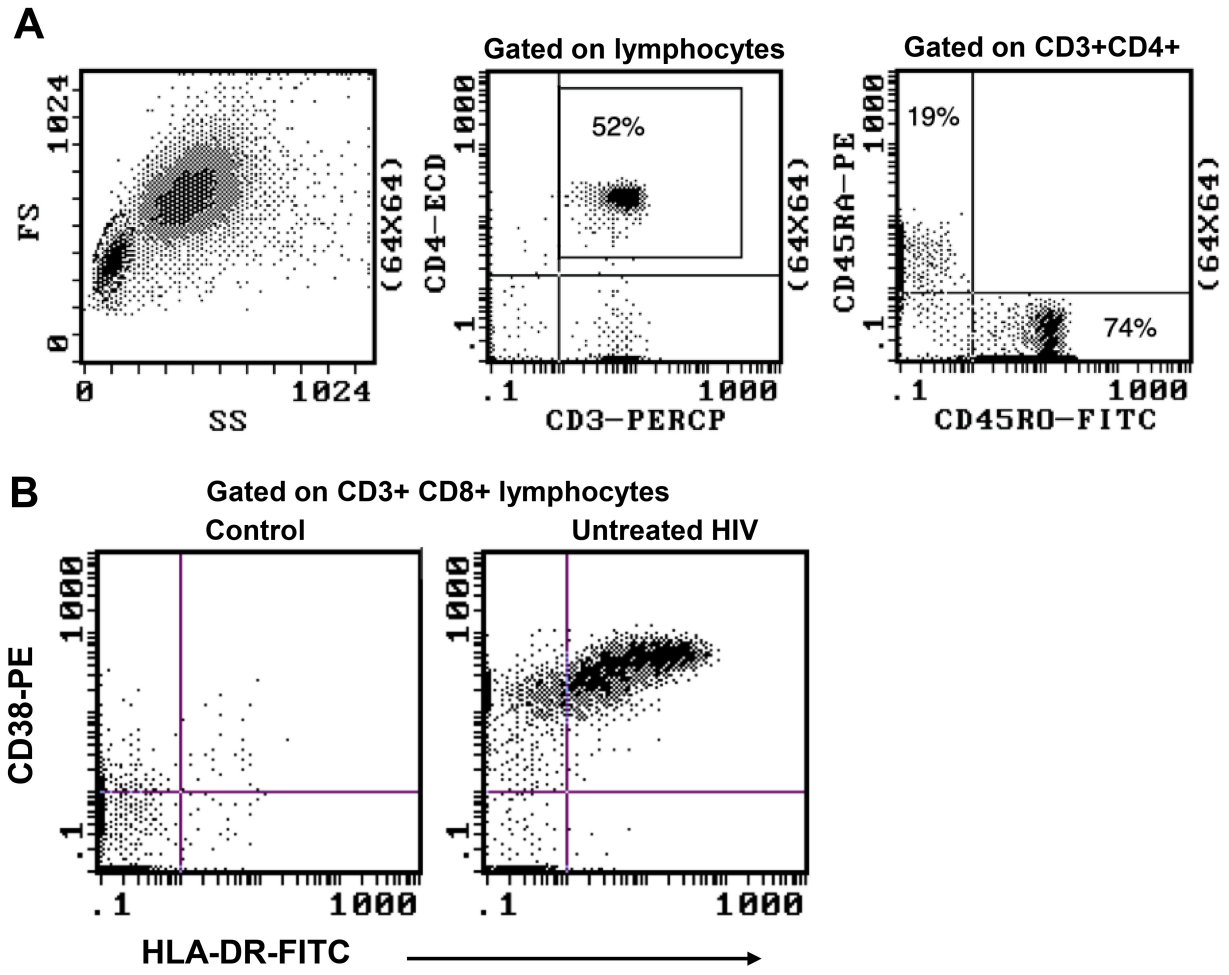
Representative flow plots from original four-colour flow cytometry performed on whole blood samples during 1995–1997. **(A)** Immunophenotype gating for CD45RA+ and CD45RO+ CD4 T cells. **(B)** Expansion of highly activated CD38+HLA-DR+ CD8 T cells during early untreated HIV-1 infection compared with an uninfected control.

**Figure 6:**
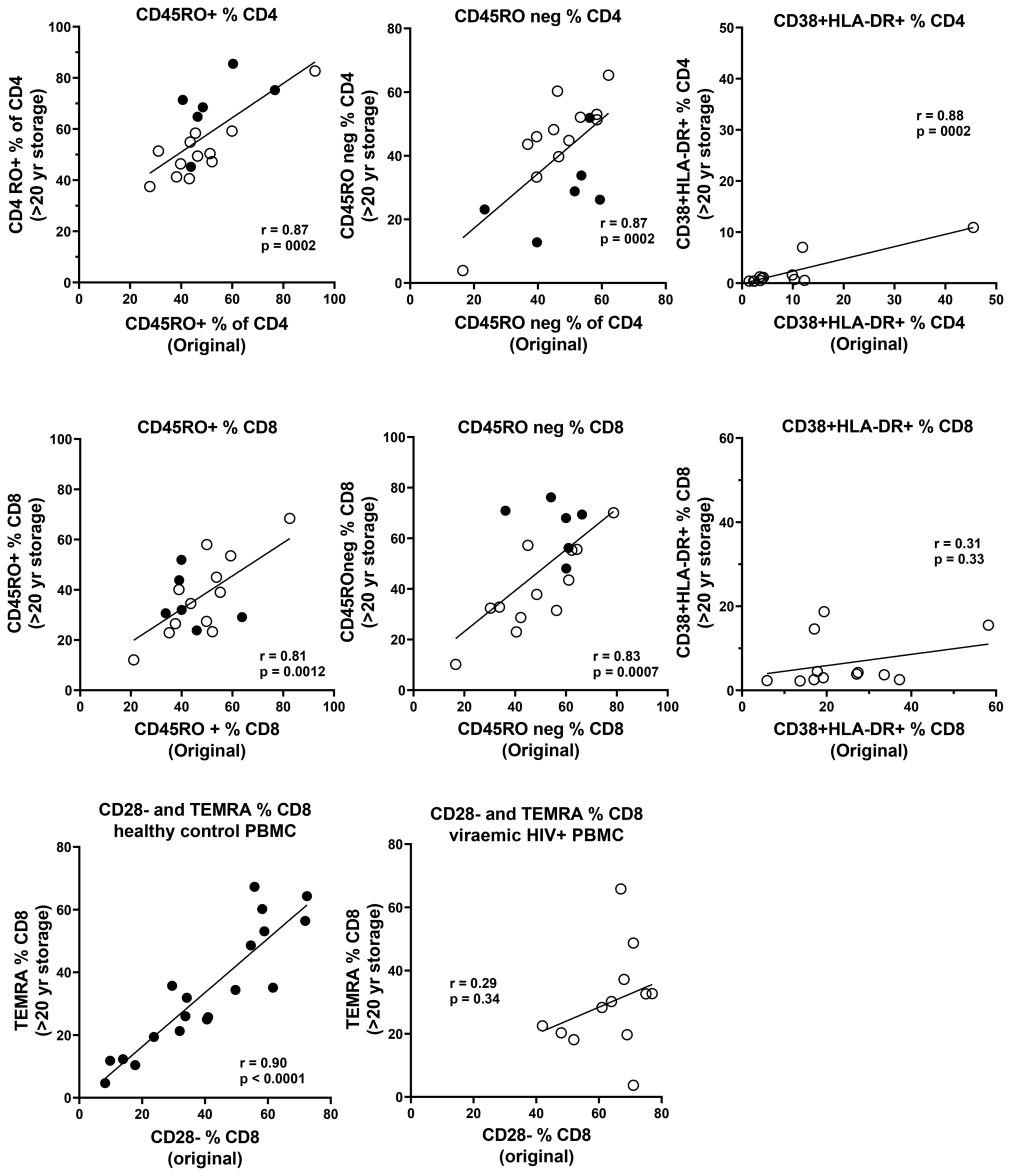
Correlations between original immunophenotyping performed in 1995–1997 in fresh blood and long-term cryopreserved PBMC separated from the same blood draw (Pearson correlation). Results for PBMC from HIV-uninfected controls are shown as solid circles, and results for viraemic HIV+ donors are shown as white circles for CD45RO+ and CD45RO negative subsets of CD4+ and CD8+ T cells. Results for CD38+HLA-DR+ activated CD4+ and CD8+ T cells are shown only for viraemic HIV+ donors. Results for CD28 negative versus terminally differentiated CD8+ T cells are shown separately for both HIV-uninfected controls and viraemic HIV+ donors.

Data were available from immunophenotyping by four-colour flow cytometry, which was performed on fresh blood taken from the same blood draws used for PBMC separation in the 1990s (18). Representative flow plots from this era are shown in **Figure 5**, including CD45RA+ and CD45RO+ CD4 T cells, and CD38 and HLA-DR co-expression in control subjects compared with those with early untreated HIV-1 infection. Correlations between the historical results and the results from thawed long-term cryopreserved PBMC are shown in **Figure 6**. In HIV+ samples, we observed strong correlations for naïve and memory subsets in both CD4 and CD8 T cells. However, there was a clear loss of activated CD38+ HLA-DR+ subsets of CD4 and CD8 T cells after cryopreservation for these untreated viraemic HIV+ patients, although there was still a correlation with the original results. Similarly, the CD28−/TEMRA phenotype of CD8 T cells appeared to be retained after cryopreservation in both healthy control and HIV+ patient PBMC but appeared to be better preserved in healthy control PBMC.

### Antigen-specific T-cell responses after long-term cryopreservation

**Figure 7:**
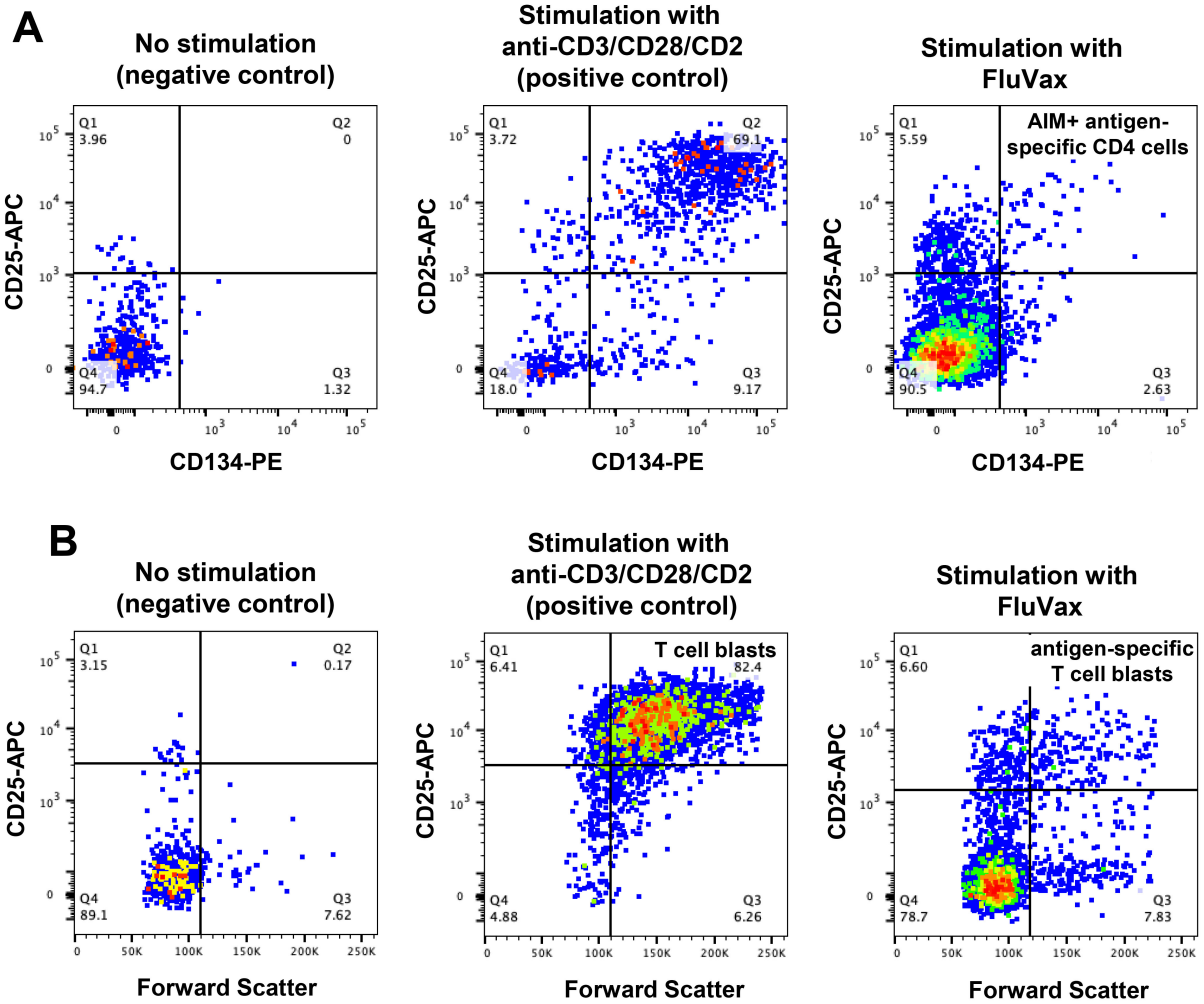
Antigen-specific CD4 T-cell function assays in long-term cryopreserved PBMC. **(A)** Representative flow plots of mitogen- and antigen-specific CD4 T cells, gated on day 2 cultured CD3+CD4+ T cells, detected by activation induced marker (AIM) assay (defined as CD25+CD134+ cells). **(B)** Representative flow plots of proliferating mitogen- and antigen-specific CD4 T cells, gated on day 7 cultured CD3+CD4+ T cells (defined as CD25 high Forward Scatter high blast cells).

Antigen-specific and polyclonal CD4 T-cell responses were assessed in long-term cryopreserved samples from healthy control subjects and viraemic HIV+ patients. Gating for day 2 cultured antigen-specific T cells measured by the OX40 activation-induced marker (AIM) assay is shown in **Figure 7A**. Day 7 proliferation of T cells shown in **Figure 7B** was defined by a dramatic increase in CD25+ large blast cell numbers.

**Figure 8:**
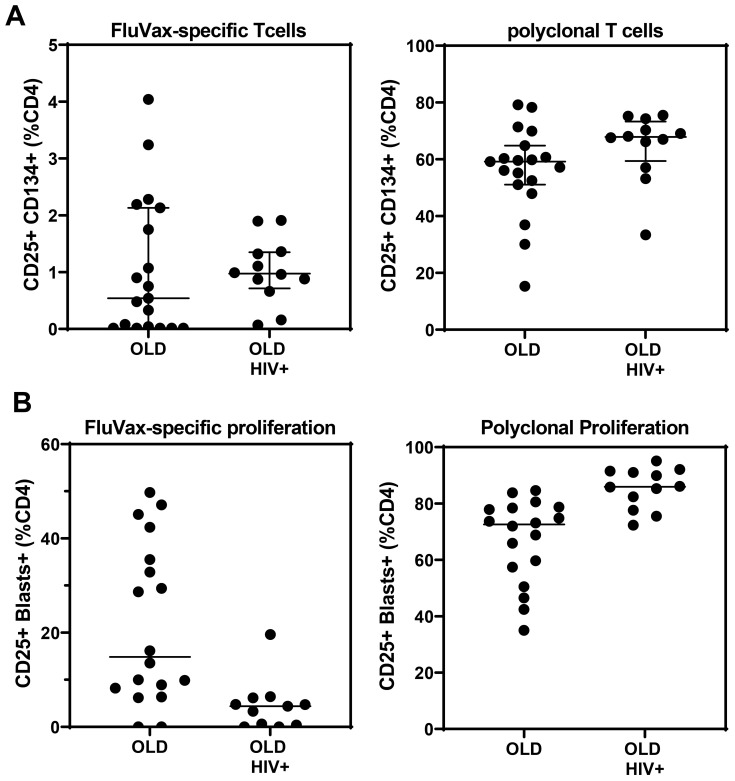
Antigen-and mitogen-specific CD4 T-cell function in >20-year cryopreserved PBMC from viraemic HIV+ patients (old HIV) and healthy controls (old), measured by day 2 AIM assay **(A)**, and day 7 proliferating CD4 T-cell blasts **(B)**.

The OX40 AIM assay (**Figure 8A**) confirmed preservation of antigen-specific metabolic signalling, showing detectable recall responses to Flu antigen in samples from 12 out of 19 control subjects and 10 of 12 HIV+ patient samples. All long-term cryopreserved PBMC retained the ability to respond to the polyclonal stimulator anti-CD3/CD28/CD2. The proportion of proliferating blasts in response to influenza antigen was reduced in the HIV+ compared with control donors (**Figure 8B**), whereas a larger proportion of cells from HIV+ patients were responsive to polyclonal stimulation measured in day 7 cultures. This proportional difference in response between HIV+ and control groups was observed in our original ^3^H-Thymidine incorporation assays in the 1990s (32). Furthermore, the OX40 AIM assay results at day 2 correlated with proliferation results at day 7 for each sample (r=0.79, p<0.001), as we previously reported for fresh PBMC (21).

**Figure 9:**
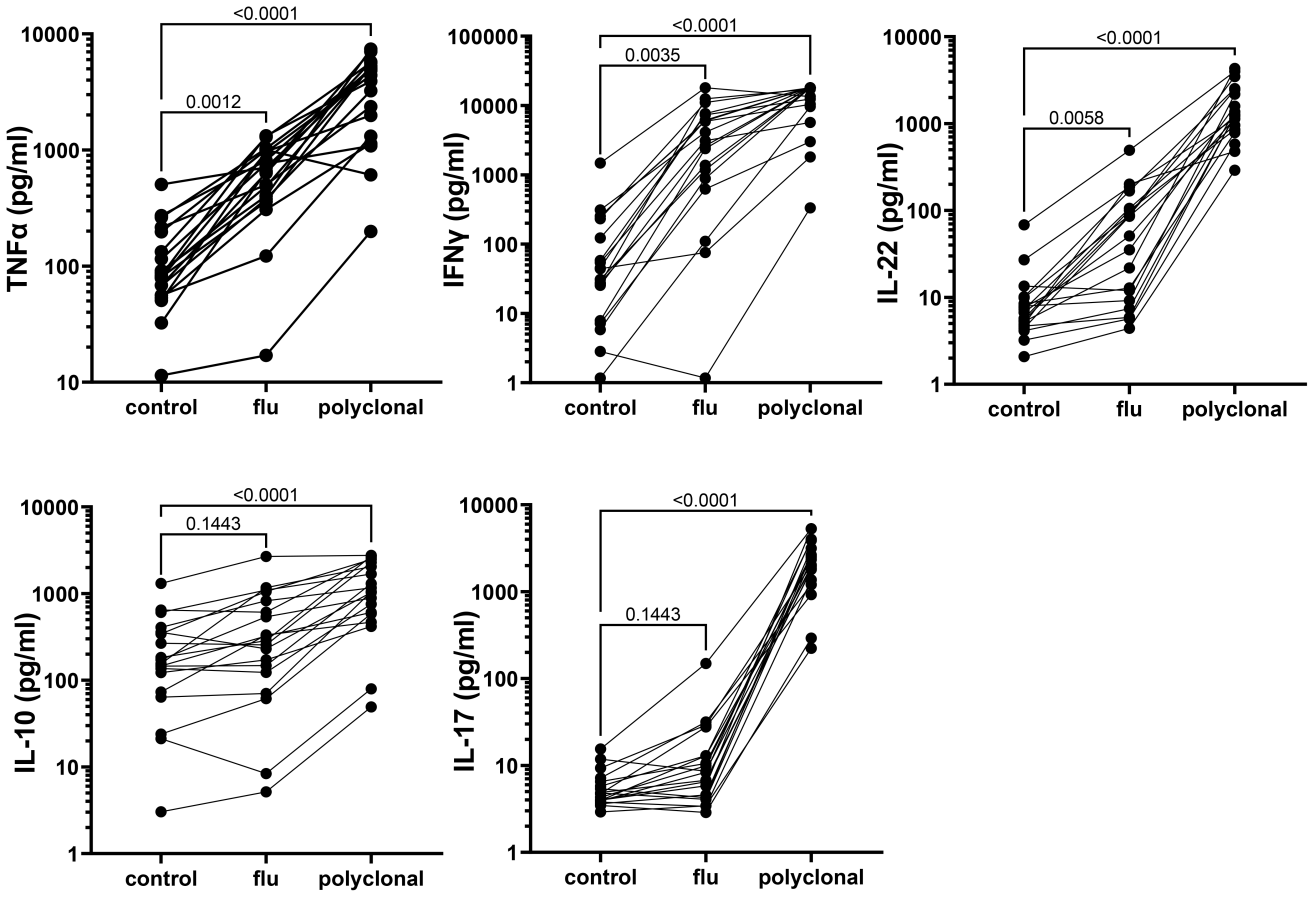
Cytokine response to antigenic and polyclonal stimulation, measured in 48-h culture supernatants from long-term cryopreserved control donor PBMC (Friedman Test p values).

Strong cytokine responses to antigenic and polyclonal stimulation were detected in culture supernatants collected on day 2 from the long-term cryopreserved control donor PBMC (**Figure 9**); culture supernatants from HIV+ patient PBMC were used for live virus isolation and therefore not assessed for cytokines. Polyclonal stimulation with anti-CD3/CD28/CD2 induced significant release of all cytokines tested. Influenza antigen induced a significant TNFα, IFNγ, and IL-22 response, whereas the IL-10 and IL17 response was weak (**Figure 9**).

### HIV RNA transcripts from proliferating T cells

**Figure 10:**
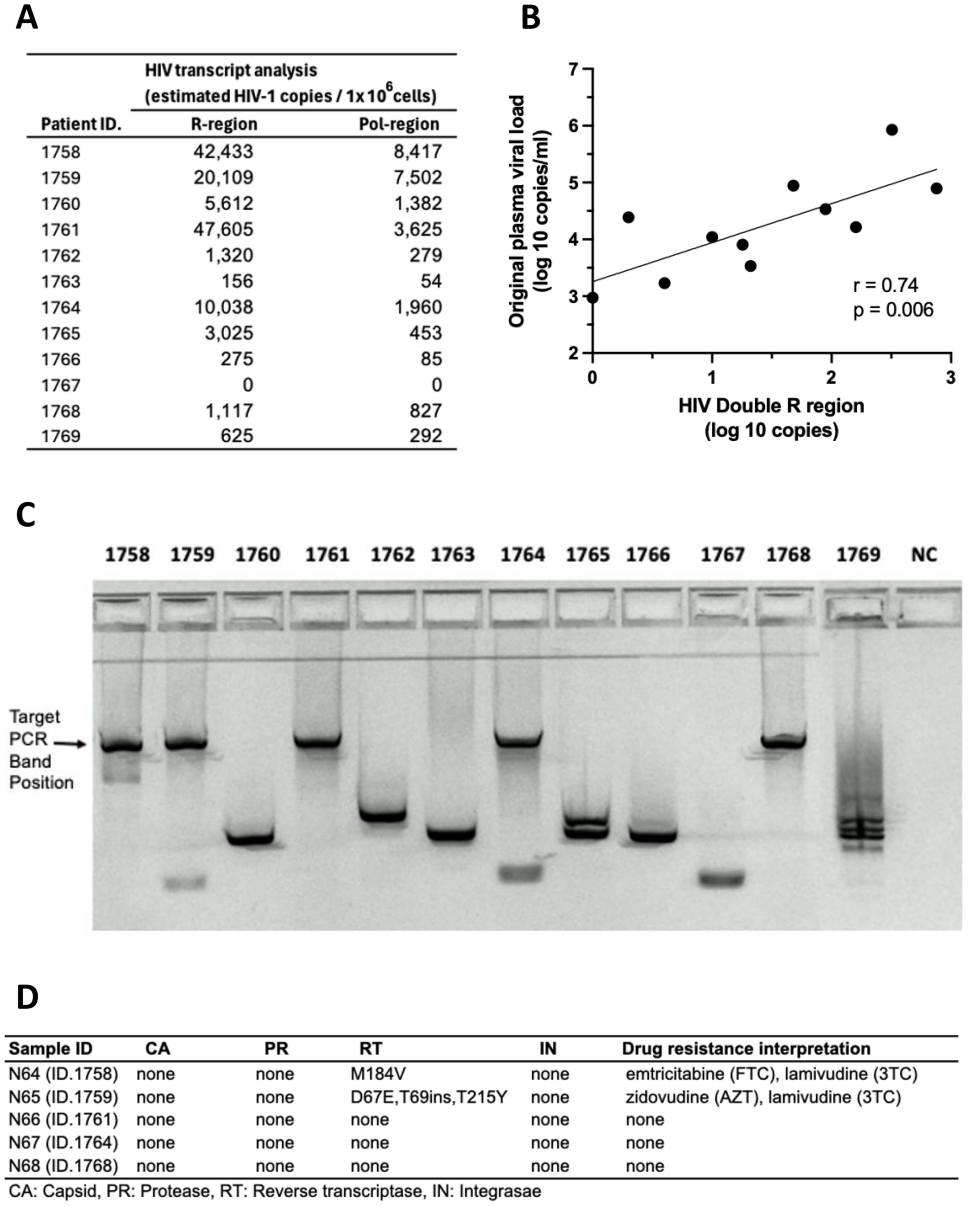
Recovery of HIV RNA after >20-year storage: activated HIV+ donor PBMC stimulated with anti-CD3/CD28/CD2. **(A)** HIV RNA transcripts in the R and pol regions. **(B)** Correlation between levels of HIV RNA “Double R” transcripts from activated PBMC and original plasma viral loads from 1995 to 1996. **(C)** Amplification of long HIV transcripts. **(D)** Presence of sequence mutations in amplicons from long HIV transcripts.

PBMC cultures from HIV+ donors, stored for >20 years, were activated for 7 days with the T cell mitogen, anti-CD3/CD28/CD2, and intracellular HIV-1 RNA expression is shown in **Figure 10A**. HIV-1 transcripts were detected in 11 out of 12 PBMC samples following activation *in vitro*. For the 11 samples that had available plasma viral loads from the original blood samples, there was a significant correlation between activation-induced HIV-1 RNA and the original plasma viral load levels (**Figure 10B**).

Furthermore, amplicons were generated from long unspliced transcripts spanning at least 4 kbp, from intracellular HIV RNA from 5 out of 12 PBMC cultures from HIV+ subjects (**Figure 10C**). Nanopore sequencing demonstrated wild-type unspliced HIV-1 transcripts up to and including pol gene, consistent with replication competent viral transcripts. Also, sequences of HIV-1 transcripts recovered from cell-free culture supernatants matched the wild-type cellular transcripts (**Figure 10D**).

## Discussion

This study is novel in directly comparing T-cell phenotyping between the original fresh blood data and in PBMC from the same samples after decades in cryogenic storage, performed by the same flow cytometry laboratory. The principal outcomes of this study were preservation of all major PBMC subpopulations and the majority of T-cell subsets, and preservation of antigen-specific and polyclonal T-cell responses in both viraemic HIV+ patients and control donors, verified by data generated decades ago from these samples (18, 32). Robust antigen-specific OX40 upregulation is not only an important indicator of preserved metabolic signalling but also essential in regulating T-cell clonal expansion and survival, and effector cell differentiation (33–35). We observed a robust cytokine response to antigenic and polyclonal stimulation in long-term cryopreserved PBMC. The IL-2 response was not assessed; however, we observed large clumps of T-cell blasts in culture, confirmed by high proportions of large CD25++ lymphoblasts in day 7 antigen-specific cultures, which therefore may indicate that IL-2 release was substantial. These observations provided a snapshot of proliferating cells, which is different to tracking proliferation by the CFSE assay. Some of the T-cell subsets were only recently described (e.g., MAIT cells, and CXCR5+ CD4 and CD8 T cells); therefore, their representation in long-term cryopreserved PBMC provided a unique retrospective analysis in these rare patient cohorts. We also demonstrated the ability to recover HIV RNA from cultured PBMC after 27 years of cryopreservation. Overall, our study provides crucial evidence and rationale for ongoing maintenance of long-term biorepositories, to facilitate retrospective cutting-edge immunovirology research decades after sample collection.

In general, the observed differences in leukocyte populations and T-cell subsets between recent and long-term cryopreserved PBMC primarily reflected known effects of age-associated and HIV infection-associated effects on phenotypes. Our previous studies included relatively older subjects infected with *nef*-deleted HIV and their age-matched uninfected controls (18), whereas the current study compared PBMC from these older controls versus a separate cohort of highly viraemic HIV+ subjects (17). Our recently cryopreserved PBMC and fresh PBMC were not age-matched to these archived PBMC samples. Donor age effects may include reduced CD4 T cells, increased naïve but reduced memory and plasma B cells, reduced plasmacytoid dendritic cells (26), and reduced CD56-bright NK cells (36). Changes in leukocyte populations in long-term cryopreserved PBMC not reported to be donor age-associated included increased CD8 T cells, reduced total NK cells (36), increased myeloid dendritic cells (26), and reduced CD16+ monocytes (28, 29). For example, non-classical CD16+ monocytes are reported to be increased in the elderly (29); however, our results suggested that they are susceptible to loss in long-term cryopreservation.

Well-known phenotypic changes associated with HIV infection, including increased proportions of CD8 T cells and plasmablasts, and reduced proportions of basophils, dendritic cells, NK cells, monocytes, and B cells, particularly the memory B-cell subset, were observed in the thawed HIV+ PBMC, as expected. HIV-1 infection characteristically induces T-cell activation markers, particularly upregulated expression of CD38 and HLA-DR, and loss of CD28 on circulating CD8 T cells (30, 31). We demonstrated correlations between the proportion of activated CD38+HLA-DR+ T cells and terminally differentiated CD28-negative CD8 T cells (equivalent to CD8+ TEMRA) in HIV+ patient PBMC and the corresponding original flow cytometry results, but absolute numbers of activated T cells were significantly reduced in the cryopreserved PBMC. The proportion of activated cells is related to the degree of viral replication, which is elevated during early primary infection (37), decreased as a result of antiretroviral therapy (38–40), and often normalised in Elite Controllers with undetectable plasma viral loads (18, 41). The activated cells are poised to undergo spontaneous apoptosis during short-term culture *in vitro*, associated with reduced expression of the IL-7R alpha chain (CD127) and reduction in Bcl-2 expression (37, 42). Therefore, not unexpectedly, there was apparent reduced survival of such activated CD38^+^HLA-DR^+^ T cells after very long-term cryopreservation, when directly compared with their observed levels at the time of blood collection (18). Importantly, HIV-specific T cells are included in these activated subsets (37, 43), such that loss of these cells may affect retrospective studies of HIV-specific T-cell immunity that rely on cryopreserved specimens. Similarly, antigen-specific T cells are found in activated T-cell subsets during acute infection, with pathogenic EBV (44), Ebola (45), and SARS-CoV-2 (46), and following immunisations with vaccinia virus and yellow fever inoculations (47, 48).

A recent study of healthy donors demonstrated decreased recovery of CD38^+^HLA-DR^+^ CD4 T cells after only 6 months of cryopreservation (12). Healthy control subjects in our study had similar levels of activated CD38^+^HLA-DR^+^ T cells in fresh blood and similar reductions after >20 years. However, we demonstrated proportionally greater post-thaw reductions in CD38+HLA-DR+ CD4 and CD8 T-cell subsets in viraemic HIV+ patients. If these same PBMC had been thawed and phenotyped again soon after cryopreservation, it is likely that a loss of activated CD38+HLA-DR+ T cells may have also been observed within a short cryopreservation time (12), thereby confirming the effect of cryopreservation time vs. the cryopreservation process as the reason for loss of HIV-activated T cells. We were not able to provide a definitive answer to this question as we did not currently have access to viraemic HIV+ patient blood samples. How these cells failed to survive, whether the cells were lost during the freezing or thawing steps or even during cryogenic storage, remains unclear. Apoptosis of activated T cells from HIV+ PBMC can be ameliorated by treatment with the cytokines IL-2 or IL-15 (42, 49), but whether their addition before cryopreservation improves phenotypic and functional preservation requires further study. CD38+HLA-DR+ T cells were not found outside the standard lymphocyte gate in a region consistent with lower forward scatter/higher side scatter typical of dead cells (42), nor were they overrepresented in the non-viable stained cell gate (not shown). Neither were these activated cells lost in clumps of dead cells during thawing, because we used DNase treatment to prevent clumping secondary to DNA release from dead or damaged cells. We therefore assume that most of the activated cells from viraemic donors experienced complete physical deterioration during thawing and processing.

Cryopreserved PBMC from HIV+ donors have been widely used for enumeration of antigen-specific T cells (50) or measurement of the proviral reservoir within activated CD4 + T cells (51).We observed lower response rates to influenza after long-term cryopreservation, compared with a recent study where we detected influenza-specific CD4 T-cell responses in fresh blood from five out of six control donors, and in recently cryopreserved PBMC from 42 out of 45 convalescent COVID-19 patients (20). The influenza strains used in the 2018 vaccine formulation, which we used here as the antigen in the OX40 AIM assay and proliferation assay, were not circulating during the 1990s, although considerable cross-reactivity with variable and conserved epitopes against 1990s strains may be expected (52). In the absence of time travel, a definitive comparison of immune responses between recent and long-term cryopreserved PBMC from the same blood collection is not feasible.

Controls for immunovirological studies may require access to decades-old specimens; for example, immunological investigations of current COVID immunity compared with pre-SARS-2 and SARS-1 outbreaks requires a 20-year specimen history. Immunovirological analysis of HIV replication and immunopathogenesis in viraemic patients not receiving antiretrovirals is virtually impossible today, requiring access to 30-year archival specimens. A well-maintained biorepository can facilitate retrospective analyses of newly described leukocyte subsets. For example, mucosal-associated invariant T (MAIT) cells, described relatively recently (53), are effector-memory CD4 and CD8 T cells with pro-inflammatory cytotoxic phenotypes, exhibit pleiotropic functions, and can represent surprisingly large proportions of CD8 T cells in some individuals. Long-term outcomes related to MAIT cells are reliant on retrospective analysis of older biorepositories. However, our results suggest that these cells may not be well preserved in all PBMC samples, as they were particularly reduced in HIV+ donors (54). This finding may also be an effect of age disparities between the study populations (55).

This study supports the feasibility of using long-term archived PBMC to directly compare CD4+ T-cell subset-specific levels of inducible HIV RNA production from the PBMC proviral reservoir before and after many years of treatment. Although we observed significant losses in activated CD38+HLA-DR+ T-cell subsets after cryopreservation, the virus recovered from these cells would primarily represent expansions of the predominant strain at that time (51). For a complete representation of viral evolutionary history in an individual patient, long-term resting T-memory subsets would be preferred, for which we demonstrated high rates of preservation.

Our results demonstrate that virus within infected CD4 T cells can be reactivated after decades of storage, facilitating detailed longitudinal studies of HIV DNA reservoirs in infected CD4 T cells. Highly potent ART was commenced nearly 20 years ago, so study of pretreatment PBMC would be important to understand the establishment of the long-term reservoir. The ability to combine analysis of antigen-specific T cells together with specific subsets, plus activation of proviral HIV, will allow detailed analysis of HIV reservoirs before and after antiretroviral therapy. We have previously described HIV DNA measurements in resting versus activated CD4+ T-cell subsets (51, 56) and in antigen-specific CD4+ T cells (57). However, most of this DNA is defective (58) whereas analysis of HIV RNA recovery from such subsets, especially from cryopreserved PBMC, has so far been quite limited (25, 59, 60). Previous studies using quantitative viral outgrowth assays suggest that only one in a million CD4 T cells may contain replication competent proviral HIV-1 DNA, although assays of intact proviral HIV-1 DNA suggest a 10–100-fold higher rate of replication competent provirus infection (61). These assays may be hampered by inefficient reactivation of latently infected cells (61). However, our current and a previous study (25) both confirm that anti-CD3/anti-CD28/anti-CD2 is a very effective reagent when combined with the Double R assay, and therefore viral reactivation studies are still feasible when only limited quantities of archived PBMC are available.

This study was limited by the unavailability of age-matched donor specimens. Donors for the long-term cryopreserved control PBMC (18) were significantly older than the untreated HIV+ patients (17) and the known donors for the recently cryopreserved PBMCs. Therefore, proportional differences in various leukocyte populations were not necessarily associated with PBMC storage duration but confounded by known differences in donor age. Furthermore, current clinical management of HIV+ patients meant it was impossible to obtain recently cryopreserved PBMC from viraemic untreated HIV+ patients to compare with long-term cryopreserved samples. To address these limitations, a major strength of this study was flow cytometry performed on long-term cryopreserved PBMC in the same laboratory that performed fresh whole blood flow cytometry in the 1990s, using equivalent gating protocols. However, as discussed above, an ideal comparison would also have included immunophenotyping performed on these PBMC soon after cryopreservation in the 1990s. Apart from activated T-cell subsets in HIV+ patients, which appear to be lost early in the cryopreservation process (12), our observed correlations between the original fresh blood results and from PBMC stored at the same time from the same blood collection supports claims of comparable preservation in recently-described T-cell subsets.

When comparing archived PBMC samples with recent and prospective collections, other limitations and quality variables deserve careful consideration (62). Differences in blood sample handling and PBMC cryopreservation techniques between labs and changes over time may impact sample quality. Our data showed that differences in counting methods used in the 1990s confounded comparisons of cell recovery data. However, the higher-than-expected recovery combined with high viability indicates that quality of these archived PBMC was also high. Ideally, archived samples should be sourced from institutions with quality management systems to ensure appropriate staff training and adherence to optimal procedures, along with appropriate record keeping of protocol deviations. Delayed blood sample processing and poor separation techniques can result in a high level of granulocyte contamination in PBMC, which can result in gross cell clumping and loss of viable lymphocytes upon thawing. Use of DNase in our study enabled all thawed cells to remain in suspension for an accurate assessment of viability. Therefore, thawing protocols for archived PBMC should include DNase treatment. Finally, the probability that some cellular or DNA/RNA degradation will occur over time, despite careful storage practices, requires periodical assessment to determine if an archived specimen collection is fit for purpose. Our study confirms the importance and feasibility of reconciling these quality issues.

## Conclusion

This study uniquely demonstrated that diverse leukocyte populations and T-cell subsets of interest to immunovirological research can be preserved after decades of cryogenic storage. Antigen-specific responses remained detectable after decades in cryopreservation, but at reduced levels. The findings confirm that application of novel immunovirology research on stored PBMC is possible and can be conducted with confidence on long-term biorepositories of PBMC collected from various patient groups, including HIV+ donors, subject to confirmation of quality parameters including viability and subset representation, as described here.

## Data Availability

The datasets presented in this study can be found in online repositories. The names of the repository/repositories and accession number(s) can be found below: PRJNA1088911 (SRA).
