# Supplementary material for: Preservation of functionality, immunophenotype, and recovery of HIV RNA from PBMCs cryopreserved for more than 20 years
Source: Front Immunol. 2024 Aug 16;15:1382711. doi: 10.3389/fimmu.2024.1382711 (PMC11361978; doi:10.3389/fimmu.2024.1382711)
Supplement: Supplementary file 1 [file Table1.docx]

**Supplementary Table 1** – Monoclonal antibodies used in this study.

| **Panel** | **mAb/reagent** | | **Clone** | | **Fluoro-chrome** | | **Supplier** | |
| --- | --- | --- | --- | --- | --- | --- | --- | --- |
| **PBMC subsets** | Zombie AQUA live/dead | |  | | BV510 | | BioLegend | |
|  | CD45 | | 2D1 | | APC-H7 | | BD | |
|  | CD56 | | NCAM16.2 | | APC | | BD | |
|  | CD16 | | 3G8 | | BV421 | | BD | |
|  | CD19 | | SJ25C1 | | BV711 | | BD | |
|  | CD3 | | SK7 | | PerCP-Cy5.5 | | BD | |
|  | CD4 | | L3T4 | | BUV496 | | BD | |
|  | CD8a | | SK1 | | BUV805 | | BD | |
|  | CD27 | | M-T271 | | APC-R700 | | BD | |
|  | IgD | | IA6-2 | | BV605 | | BD | |
|  | HLA-DR | | L243 | | FITC | | BD | |
|  | CD38 | | HIT2 | | BUV496 | | BD | |
|  | CD14 | | MøP9 | | PE | | BD | |
|  | CD11c | | Bly6 | | PE-Cy5 | | BD | |
|  | CD123 | | 7G3 | | BV786 | | BD | |
| **T cell subsets** | Zombie AQUA live/dead |  | | BV510 | | BioLegend | |  |
|  | CD3 | | SK7 | | PerCP-Cy5.5 | | BD | |
|  | CD4 | | L3T4 | | BUV496 | | BD | |
|  | CD8a | | SK1 | | BUV805 | | BD | |
|  | CD45RA | | HI100 | | BUV737 | | BD | |
|  | CD38 | | HIT2 | | BUV496 | | BD | |
|  | HLA-DR | | G46-6 | | BV711 | | BD | |
|  | CD27 | | M-T271 | | APC-R700 | | BD | |
|  | CXCR3 | | REA232 | | Biotin | | Miltenyi | |
|  | Anti-Biotin | | Bio3-18E | | VioBright515 | | Miltenyi | |
|  | CCR6 | | G034E3 | | BV421 | | BioLegend | |
|  | CD161 | | 191B8 | | PE-Vio770 | | Miltenyi | |
|  | CXCR5 | | J252D4 | | PE-CF594 | | BioLegend | |
|  | CD127 | | HIL-7R-M21 | | BV786 | | BD | |
|  | CD25 | | M-A251 | | PE-Cy5 | | BD | |
|  | Integrin ß7 | | FIB04 | | BV605 | | BD | |
|  | CD49d | | 9F10 | | APC | | BD | |
|  | CCR5 | | REA245 | | PE | | Miltenyi | |
| **CD4 OX40 AIM assay** | CD3 | | SK7 | | PerCP-Cy5.5 | | BD | |
|  | CD4 | | L3T4 | | BUV496 | | BD | |
|  | CD25 | | 2A3 | | APC | | BD | |
|  | CD134 | | L106 | | PE | | BD | |
| **CD4 CD25^hi^ blasts assay** | CD3 | | SK7 | | PerCP-Cy5.5 | | BD | |
|  | CD4 | | L3T4 | | BUV496 | | BD | |
|  | CD25 | | M-A251 | | PE-Cy5 | | BD | |
